# Carbon Nanomaterial Doped Ionic Liquid Gels for the Removal of Pharmaceutically Active Compounds from Water

**DOI:** 10.3390/molecules24152788

**Published:** 2019-07-31

**Authors:** Carla Rizzo, Salvatore Marullo, Nadka Tz. Dintcheva, Francesca D’Anna

**Affiliations:** 1 Dipartimento di Scienze e Tecnologie Biologiche Chimiche e Farmaceutiche, Università degli Studi di Palermo, Viale delle Scienze, Ed. 17, 90128 Palermo, Italy; 2 Dipartimento di Ingegneria, Università degli Studi di Palermo, Viale delle Scienze Ed. 8, 90128 Palermo, Italy

**Keywords:** ionic liquids, supramolecular gels, graphene, carbon nanotubes, wastewater treatment, pollutant adsorption

## Abstract

Due to large drug consumption, pharmaceutically active compounds (PhACs) can be found as water contaminants. The removal of PhACs is a significant issue, as they can easily overtake traditional purification methods. Because of their surface properties, carbon nanomaterials are among the most efficient materials able to adsorb PhACs. However, their limitation is their recovery after use and their possible leakage into the aquatic system. Consequently, new hybrid supramolecular ionic liquid gels (HILGs) have been designed for the adsorption of some antibiotic drugs (ciprofloxacin and nalidixic acid) from water. The chemical–physical properties of gels, such as the temperature of the gel–sol transition, morphology, and rheology, have been studied for their use as sorbents. These properties influence the gel removal efficiency of PhAC, i.e., the best system is the gel that presents weaker colloidal forces. A fast removal (RE = 51%) is obtained in 3 h for ciprofloxacin, while a slower adsorption process is observed for nalidixic acid (RE = 88% in 24 h). HILGs can be recycled up to seven cycles and regenerated. In addition, they can be used with higher concentrations or volumes of PhAC and in a realistic apparatus like dialysis membranes. These peculiarities suggest that HILGs can be competitive with more complex sorbent systems.

## 1. Introduction

The large consumption and abuse of drugs have recently highlighted, among several ethical and medical issues, the problem of water contamination due to the persistence of pharmaceutically active compounds (PhACs) in the aquatic ecosystem [1]. The main problem related to the presence of PhACs in wastewater is that their concentration is usually low. This allows PhACs to avoid common wastewater treatment techniques employed to remove organic pollutants, such as biological processes, activated carbon adsorption, ozonation, and UV photolysis/photocatalysis [2].

In addition, the chemical nature of PhAC is extremely various and further complicated by the fact that PhACs can only be partially metabolized, so parent compounds or active metabolites can also enter in wastewaters. Finally, wastewater discharged by the pharmaceutical industry may also include organic solvents, raw materials, reactants, intermediates, active pharmaceutical ingredients, and catalysts, exponentially complicating wastewater treatment [3].

The consequence of failure in wastewater treatment is the detection of PhACs in surface waters, groundwater, or tap/drinking water in several parts of the world [4]. Therefore, the search for alternative treatment methods to effectively remove PhACs and preserve the quality of drinking water is urgent.

Adsorption is the most promising technique employed to overcome the above issue, thanks to the right balance among pollutant removal efficiency, design and operation flexibility, economic convenience, and environmentally friendliness. Nevertheless, the choice of an appropriate adsorbent is still rarely investigated, and an alternative to the most widely used activated carbon has to be found. Indeed, while activated carbon displayed efficient removal for hydrophobic pharmaceuticals, it showed inefficient removal for electrically charged or hydrophilic pharmaceuticals [5]. In addition, the working capacity of activated carbon greatly decreases in the presence of natural organic matter, and the regeneration of these adsorbents is difficult [6]. Good alternatives seem polymeric adsorbents such as synthetic resins [6], magnetic nanosorbents [7], molybdenum disulfide nanosheet [8], nanoscale zeolites [9], and, above all, carbonaceous nanomaterials [10].

In particular, carbon nanotubes (CNTs) and graphene have been extensively applied for the adsorptive removal of PhACs from the aqueous phase. These materials show excellent performance because of their surface properties (like a large specific surface area) and the presence of specific functional groups on the adsorbent surface. The high hydrophobicity and mechanical properties of graphene and CNTs further enhance their PhACs’ adsorption capabilities. For example, the interaction among some PhACs and several carbonaceous nanomaterials demonstrated the best adsorption capacity of graphene [10]. However, the adsorption process is frequently influenced by several factors, which include pH and the possible presence of other organic materials. In addition, the high cost of the material and the environmental impact of the possible dispersion of these nanomaterials in the aquatic ecosystem, up to now, have represented the biggest limitations to their real application.

The immobilization of the nanomaterials in a matrix, maintaining their unique adsorption properties, is a great challenge and may overcome the issue of their undesired release in the aquatic environment. For this reason, graphene–hydrogels beads have been recently employed for PhAC removal [11]. In particular, hydrogels showed an excellent adsorption capacity for ciprofloxacin, a largely used antibiotic, thanks to their combined interactions favoring adsorption mechanisms. Moreover, reducing the size of the hydrogels can significantly accelerate the adsorption process and enhance the removal efficiency of pollutants from an aqueous solution. More recently, it has also been demonstrated how the water content of these gels is important in the adsorption process of several water pollutants [12]. In addition, biochar, a heterogeneous carbonaceous material dispersed in a chitosan/humic acid hydrogel, has been used as an efficient adsorbent for ciprofloxacin-contaminated water. This hybrid gel can also be reused for four cycles [13]. These findings open the way to the application of hybrid gels as promising adsorbents for the removal of antibiotic pollutants from aqueous solutions.

Even though the application of supramolecular gels for this purpose has not been explored, these supramolecular gels could be an alternative to polymeric gels for the incorporation of carbon nanomaterials. Indeed, supramolecular gels are formed by low molecular weight molecules that aggregate in a 3D network held by supramolecular interactions. The 3D network can trap high amounts of a solvent [14,15] and, as a consequence, gels are recognized as organo-, hydro-, or ionic liquid gels [16]. These gels, thanks to the presence of non-covalent interactions, can easily adsorb several species, and for this reason, they have been recently applied to the removal of water pollutants, such as metal ions and dyes [17]. Thus, the inclusion of highly efficient sorbents that can exert multiple interactions with pollutant species (such as carbon nanomaterials) in supramolecular gels can solve the above issue. On the other hand, the use of supramolecular gels could represent a further advantage if gels formed in an ionic liquid (IL) solution, so called ionic liquid gels (ILGs), are considered. Indeed, these gels could conjugate the already known ability of ILs to disperse carbon-based materials, with their high solubilizing ability, which could avoid issues due to their rare affinity to the hydrophilic or charged pharmaceuticals generally detected for activated carbon. ILGs have recently proven to be good adsorbents for dye removal [18]. Consequently, in this work, the performance of hybrid ionic liquid gels (HILGs) has been tested for the removal of PhACs from wastewater. To the best of our knowledge, this is one of the few studies in which ILGs and, in particular, HILGs have been used for this purpose.

**Scheme 1:**
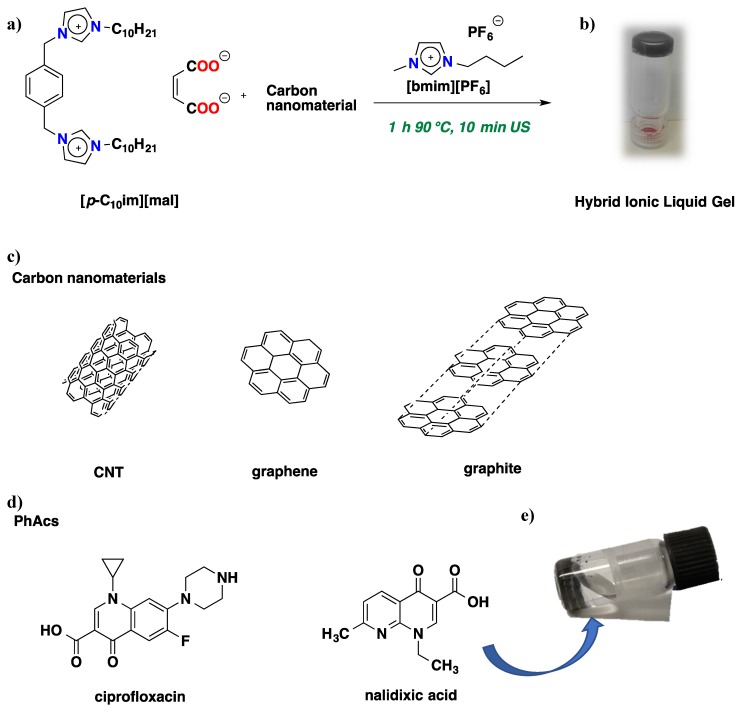
(**a**) Schematic representation of hybrid supramolecular ionic liquid gel (HILG) preparation, including the structure of the gelator and IL, (**b**) a photo of the HILG, (**c**) the structure of the carbon nanomaterials used, (**d**) the structure of pharmaceutically active compounds (PhACs) used as water pollutants to test the adsorption capacity of gels, (**e**) and the PhAC water solutions cast on the HILG.

The ionic liquid gel used is formed by an imidazolium dicationic organic salt coupled with a maleate anion, **[*p*-C_10_im][mal]**. This organic salt is able to gel ionic liquids such as 1-butyl-3-methyl imidazolium hexafluorophosphate, **[bmim][PF_6_]** (Scheme 1). The performance of the gel in terms of material properties and PhAC adsorption capacity have been tested in the presence of carbon nanomaterials that have been efficiently applied to the removal of PhAC from wastewater, like single-walled carbon nanotubes (CNTs), graphene, and graphite (Scheme 1c). The last one was selected for its low cost and for comparison with nanomaterials.

Gelation tests, the temperature of the gel–sol transition, morphology by means of scanning electron microscopy (SEM) or polarized optical microscopy (POM), and rheology were used to understand the properties of the HILGs. Their capacity for PhAC uptake from wastewater was investigated, using solutions of common antibiotics, such as ciprofloxacin and nalidixic acid (Scheme 1d). The removal efficiency of different gels, the kinetic of adsorption, the recycle of the gels, and the influence of PhAC concentration and volume were considered to assess the performance of HILGs as sorbent systems.

Notably, the best system showed a fast uptake of ciprofloxacin reaching the adsorption equilibrium in just 3 h and a higher removal efficiency for nalidixic acid. HILGs can be reused for several adsorption cycles, regenerated, and reused. Moreover, they can be used with different concentrations or volumes of PhAC solutions, opening their application for real water treatment methods.

## 2. Results and Discussion

### 2.1. Gel Preparation and Thermal Behaviour

Both pristine and hybrid gels were prepared using **[*p*-C_10_im][mal]** as a gelator and **[bmim][PF_6_]** as a gelation solvent. For simplicity, gels will henceforth be indicated as **pristine-G** or **carbon material-G** (**CNT-G**, **graphene-G**, **graphite-G** depending on the material introduced in the HILGs).

To prepare HILGs, the gelator and carbon nanomaterials were previously ground to form a homogeneous solid mixture, which was subsequently dispersed in an IL solution through US irradiation and heating at 90 °C (Scheme 1a). The effect due to both the gelator and carbon material percentage was analysed.

Firstly, using a carbon material concentration equal to 0.2 wt%, the effect of changing the gelator concentration was assessed. The minimal amount of gelator needed to form the gel, i.e., the critical gelation concentration (CGC) and the gel–sol transition temperature (*T*_gel_) were determined for all the gels obtained (Table 1).

Black opaque gels, stable in the tube inversion test, were obtained after resting overnight at 4 °C (Scheme 1b), whereas white opaque gels were observed in the case of **pristine-G** (Figure S1).

Although the presence of carbon materials does not significantly influence the CGC, showing only a slight increase for hybrid gels compared with the pristine one (entries 1, 4, 7, 10), it has a positive effect on *T*_gel_, which increases by about 20 °C compared to the **pristine-G** at the same concentration. Previous reports about supramolecular interactions working on ILG formation state that ionic interactions together with hydrogen bonding are the driving forces behind the process [19,20]. Carbon materials assure higher thermal stability to the gels, likely thanks to additional supramolecular interactions, such as the π–π interactions, that can be established among the gelator and the carbon materials and, in turn, could also strengthen the IL-composite gelator interactions [21,22]. These additional interactions are able to reinforce the 3D-network of the gel, inducing a higher thermal resistance to the flow of the gel. This behaviour is in agreement with the enhanced properties observed for other hybrid supramolecular gels compared to their corresponding pristine gels [23].

On the other hand, the data in Table 1 do not allow discrimination among the hybrid gels taken into account, as *T*_gel_ differences at the same gelator concentration fall within the range of experimental error, indicating a similar contribution of the three carbon materials to the gel formation and thermal stability.

Finally, a significantly higher thermal stability for the gels can be detected in the presence of a larger amount of gelator, since (like all hybrid gels) *T*_gel_ increases of ≈15 °C passing from 4 wt% to 6.25 wt%. However, to avoid the environmental impact deriving from the use of large amount of gelator, a concentration equal to 4 wt%—giving gels with good thermal stability—was used, and the effect of carbon material concentration was investigated (Table S1). In particular, the percentage ranged from 0.1 to 0.6 wt%, and the data collected show that the most stable HILGs were obtained with a 0.2 wt% of carbon materials. Consequently, the rest of the study was conducted on HILGs with 4 wt% of gelator and 0.2 wt% of carbon material.

### 2.2. Morphology of Gels

**Figure 1:**
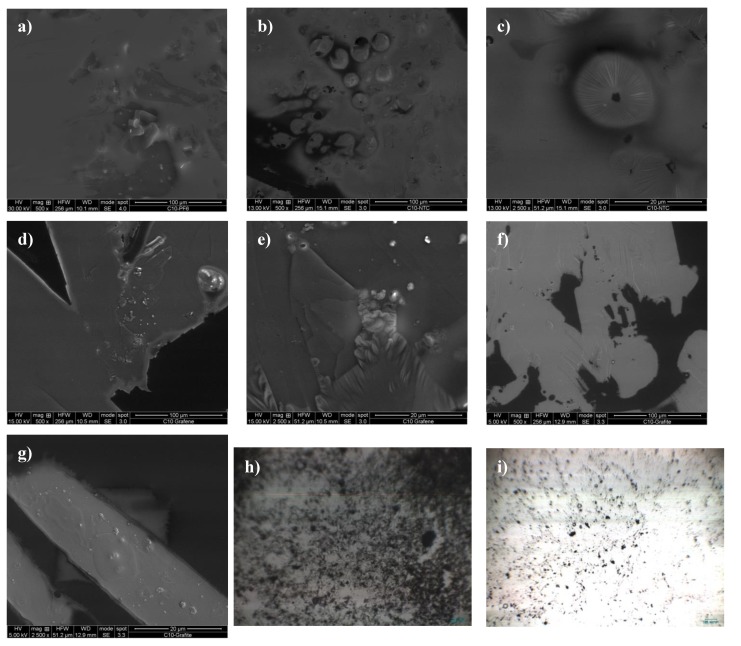
SEM images of xerogels derived from gels with 4 wt% of the gelator and 0.2 wt% of the carbon material (**a**) **pristine-G**, (**b**,**c**) **CNT-G**, (**d**,**e**) **graphene-G**, (**f**,**g**) **graphite-G**; morphologies recorded with an optical microscope (10×) (**h**) **CNT-G**, (**i**) **graphene-G**. CNT, carbon nanotube. Scale bars in (**h**) and (**i**) are 100 μm.

The morphology of hybrid gels was studied using SEM and POM. In the first case, it was possible to discriminate the different morphologies of aggregates depending on the carbon material in the gel network, while in the second case, a dispersion homogeneity was observed for the carbon material in the gel matrix (Figure 1 and Figure S2).

The morphology of the hybrid gels is significantly different compared to that of the **pristine-G** (Figure 1a), which shows a classical flat ionogel morphology, where it is difficult to observe single aggregates. On the other hand, a closer look at the hybrid gels’ morphology reveals some characteristic features of the aggregates.

In particular, spherulitic aggregates characterize **CNT-G** morphology (Figure 1b,c), indicating that the presence of CNTs induces an additional aggregation level in the self-assembled network. However, **graphene-G** and **graphite-G** show a packed “chips” morphology (Figure 1d–f), more evident in the closer view where smaller spherical aggregates can also be recognized (Figure 1e–g). Both kind of morphologies, spherulitic and chip like, are further supported by POM investigation (Figure S2). In addition, the observation of the gels under an optical microscope demonstrates that a homogeneous dispersion of the material in the gel matrix is obtained for all hybrid gels (Figure 1h,i and Figure S2).

### 2.3. Rheology of Gels

Rheological analysis of pristine and hybrid gels was performed to clearly assess the gel-like physical state of the soft materials. In particular, oscillation strain and frequency measurements were carried out on gels with 4 wt% of gelator and 0.2% of carbon materials.

**Figure 2:**
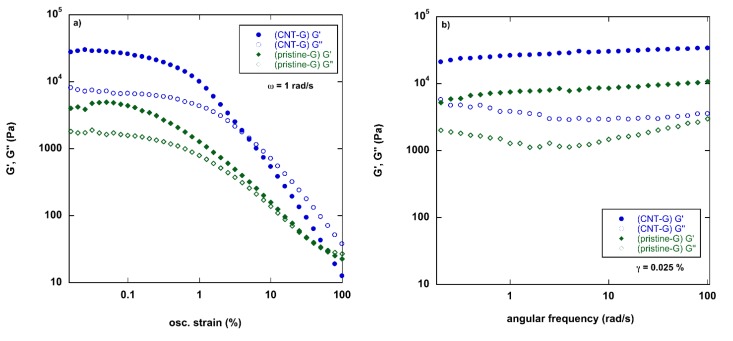
(**a**) Strain and (**b**) frequency sweeps graphs for **CNT-G** and **pristine-G**.

In all cases, the typical solid-like behaviour with a storage modulus (G′) higher than the elastic one (G′′) was observed at a low percentage of strain (γ). However, the increase of this parameter at a point named the crossover point (G′ = G′′) brings about the inversion of the moduli and clearly indicates a liquid-like behaviour (Figure 2a, Figure S3). This intermediate trend is ascribable to the gel state and is further confirmed by the fact that G′ is always higher than G′′ at a fixed strain, demonstrating that this behaviour is independent from the applied increase in the angular frequency (Figure 2b).

Trends reported in Figure 2 clearly underline better rheological performances of **CNT-G** compared to **pristine-G**. This is well evidenced by the corresponding moduli values (Table 2) and from a much lower tan δ, which is equal to the ratio among elastic and storage moduli and indicates a higher stiffness of **CNT-G** in this case.

A different scenario is observed for **graphene-G** and **graphite-G**, as their rheological responses are similar or even worse (in the case of **graphene-G)** than that of **pristine-G**. In these cases, carbon materials seem to induce a negative effect on the gels’ rheological response, forming less strong gels compared to pristine ones.

**Pristine-G** was able to be restored after mechanical or ultrasound disruption, indicating thixotropic and sonotropic properties [18]. In general, hybrid gels retain the thixotropic behavior of **pristine-G**, with the exception of **graphite-G**. However, all hybrid gels are not sensitive to ultrasound irradiation (Table S1).

The thixotropic behavior of HILGs was also assessed using rheological investigation. Unfortunately, we could not perform measurements using **pristine-G**. Indeed, as a consequence of the low *T*_gel_, the gel melted during the test. Then, we took into consideration **CNT-G** and **graphene-G** and measured the evolution of moduli at low (G′ > G′′ regimes, γ = 0.025%) and destructive (G′′ > G′ regimes, γ = 40%) strains.

**Figure 3:**
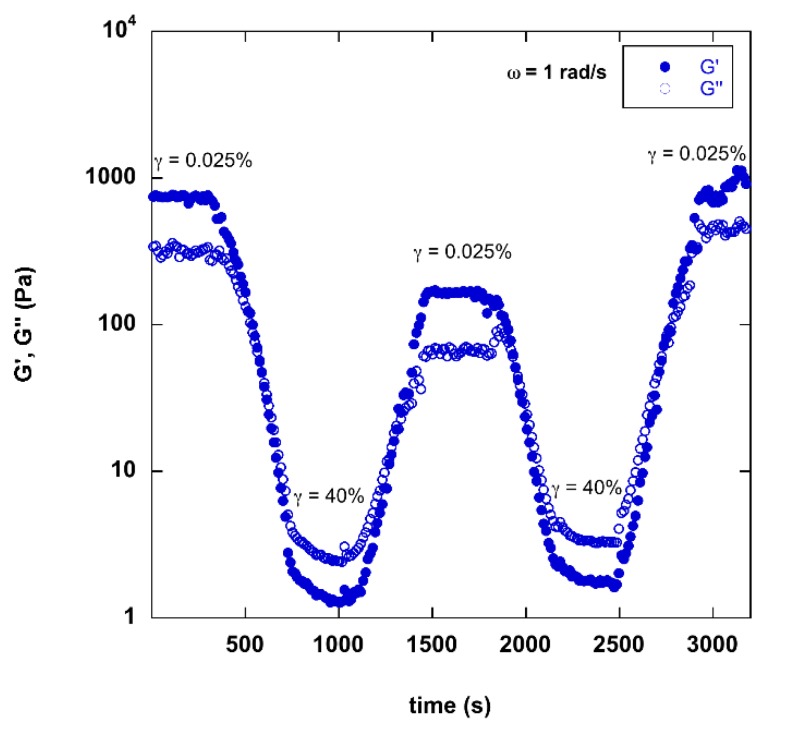
A thixotropy test on **graphene-G** at 4 wt% of the gelator and 0.2 wt% of the nanomaterial. G′ and G′′ as a function of time and application of low (G′ > G′′ regimes, γ = 0.025%) and destructive strains (G′′ > G′ regimes, γ = 40%) at 25 °C.

The percentage of strain recovery was evaluated through a comparison of the initial value of G′ with the one obtained in the LVR after disruption (Figure 3; Table S2). The data collected show that **graphene-G** exhibited a better self-healing ability than **CNT-G**, as higher percentages of recovery were calculated both in the first and the second cycles (13% and 9% for **CNT-G** and 32% and 100% for **graphene-G**, respectively). Once again, the above result can be ascribed to the different strength of supramolecular interactions working in the gelatinous network. Presumably, less strong interactions can be easily broken and quickly restored to bring the system back to the initial state.

### 2.4. PhAC Adsorption on Gels

**Figure 4:**
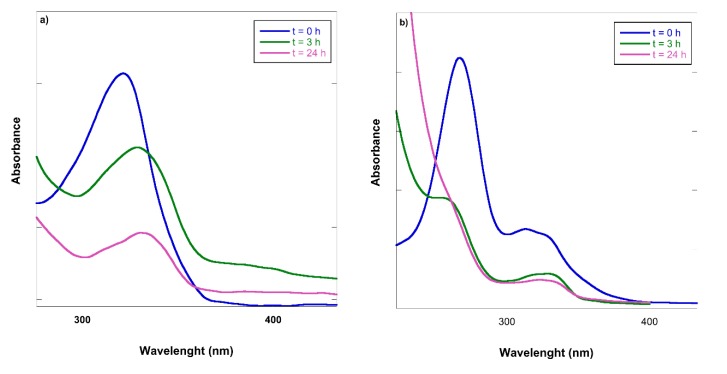
UV-vis spectra of (**a**) nalidixic acid in a water solution (1.7 × 10^−4^ M), (**b**) ciprofloxacin in a water solution (1 × 10^−4^ M), immediately after the casting of the solution (t = 0 h) and after 3 and 24 h of contact with the gel.

Nalidixic acid and ciprofloxacin water solutions at concentration of ≈10^−4^ M were used as examples of PhAC wastewater pollutants. PhAC solutions were cast on gels (Scheme 1e) and, at fixed intervals of time, were spectrophotometrically analyzed to determine the removal efficiency of materials (RE), determined through calibration curve, according to Equation (1):

RE = 100 × (C_0_ − C_i_/C_0_) (1)

where C_0_ and C_i_ represent the initial concentration and the concentration of PhAC at a given time, respectively. In both cases a decrease in absorbance values was observed after 3 and 24 h of contact with the gel phases (Figure 4).

**Figure 5:**
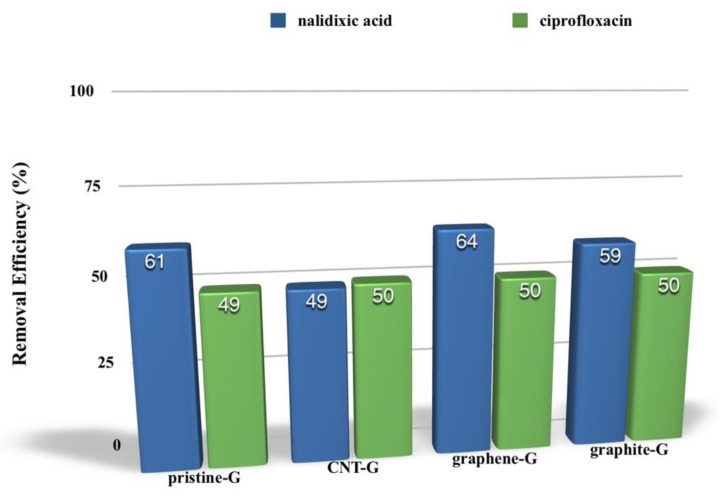
Removal efficiency after 3 h of contact of the PhAC water solution with the gel phases. Removal rate (RE) is based on triplicate runs with a reproducibility of 2%.

A fixed contact time of 3 h between the solutions and gels was chosen to screen the removal efficiency of the different gels, to ensure a homogeneous comparison. Figure 5 shows that all gels examined were able to partially remove both PhACs (Table S3). In particular, the removal rate (RE) was higher in the presence of the nalidixic acid solution than in ciprofloxacin. In addition, in the first case it is possible to discriminate the adsorption capacity as a function of the nature of the gel, and the results obtained for ciprofloxacin are almost comparable.

With the only exception of **CNT-G,** which shows a lower RE for **pristine-G**, in all other cases, the addition of carbon materials retained the same RE as the **pristine-G**. This trend is opposite to the rheological strength of the gels. Indeed, the strongest gel, **CNT-G**, possesses the lowest adsorption capacities and the weakest gel, **graphene-G**, possesses the best one. When weaker colloidal forces are active, and the gel exhibits the lowest stiffness (tan δ), the matrix is likely more able to accept PhAC molecules and reorganize around them. This also agrees well with the more pronounced self-healing ability of the above gel and is in line with some hydrogels’ adsorption capacities for metal ions [24].

In addition, the observed trend illuminates the interactions occurring among the gel matrix and carbon materials. Indeed, it has been proven that removal efficiency of PhAC increases as a function of the carbon material’s surface area and pore volume, typically in the following order: single-walled CNTs > reduced graphene oxide > multiwalled CNTs > graphene > graphite [10]. This order is reversed when materials are trapped in the gel matrix, indicating that different forces are active for the adsorption and that the hybrid gels formed soft materials with different and peculiar properties than the single components. In particular, the obtained trend seems to indicate that flatter carbon materials give rise to HILGs with better adsorption performance. This underlines that in the gel phase (unlike the shape detected in the solution), the shape of the material rather than the surface area confirms the driving parameter. In particular, 2D materials like graphene appear to be arranged better within the self-assembled network, compared to 3D materials like graphite.

**Figure 6:**
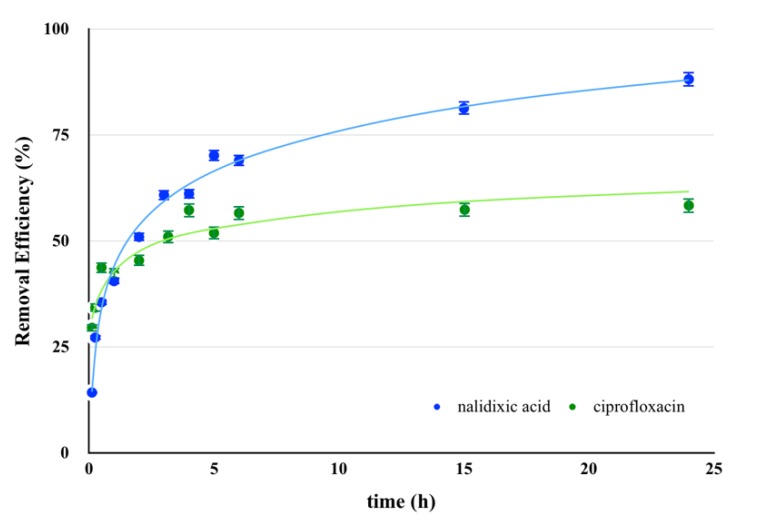
Kinetics of the removal efficiency of both PhAC water solutions using **graphene-G**. RE is based on triplicate runs with a reproducibility of 2%. Lines are drawn as a visual guide.

Because **graphene-G** had the best removal efficiency, **graphene-G** was selected as the model gel to carry out the kinetic of adsorption of both PhACs (Figure 6).

The adsorption process of ciprofloxacin is initially faster than that of nalidixic acid, as after less than 10 min, the REs are 30% and 14%, respectively. Additionally, ciprofloxacin reaches the plateau after only 3 h compared to 15 h needed for nalidixic acid (Table S4). This is extremely interesting if compared to the graphene hydrogels used for ciprofloxacin adsorption from wastewater, where the maximum of adsorption is reached in 36 h and the equilibrium is reached in 120 h [11].

Nevertheless, at equilibrium, the removal efficiency of nalidixic acid is much larger than that of ciprofloxacin (88% versus 58%). It has been previously reported that organic pollutants can be adsorbed through multiple interactions, such as the π–π electron donor-acceptor, hydrogen bonding, and hydrophobic interactions [25,26].

The π–π electron donor-acceptor interactions are especially active if graphene oxide is taken into account, as the hydrophilic moieties –OH and –COOH on graphene make it a π-electron-rich donor specie, able to accept molecules like ciprofloxacin, which has a strong electron-withdrawing ability thanks to the presence of fluorine and an N-heteroaromatic ring [11]. However, HILGs considered in this study are formed with untreated graphene, and this can explain the inferior adsorption affinity for ciprofloxacin with respect to nalidixic acid.

It is worth noting that hydrogen bonding is the main driving force between the gelator and IL to form **pristine-G**, as previously observed in similar systems [20]. Going from **pristine-G** to HILGs, the π-stacking interactions between the gelator cation and graphene also become operative. Thus, because even **pristine-G** has a good removal efficiency, it is reasonable to hypothesize that hydrogen bonding interactions can also be held among HILGs and PhACs to govern the adsorption process. On the other hand, it is well known that in the case of carbon materials, the adsorption of PhACs that have high hydrophobicity can be also driven by hydrophobic interactions established with a carbon surface or with pores [27]. Therefore, the adsorption process in HILGs can be driven by a combination of hydrogen bonding, hydrophobic interactions, and π−π interactions, mainly due to the synergetic action of the ionogel matrix and carbon nanomaterials, respectively.

The RE achieved by HILGs is competitive with respect to the ones reported for carbon nanomaterials used as sorbents for ciprofloxacin (Table 3). Better results have been obtained with graphene oxide (GO) alone or trapped in a hydrogel matrix, but after a longer adsorption time or in basic pH conditions. In addition, it is worth remembering that carbon materials used in HILG are untreated, so they are less expensive and more easily available than functionalized ones.

**Figure 7:**
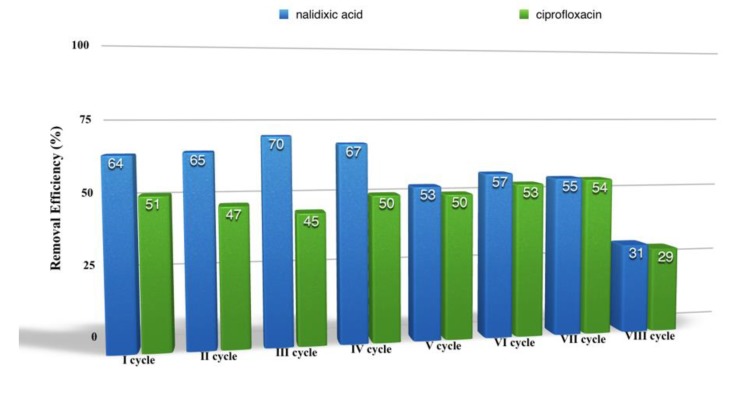
Removal efficiency of both PhAC water solutions at 3 h using **graphene-G** for recycling tests. RE is based on triplicate runs with a reproducibility of 2%.

The recyclability of **graphene-G** has been tested by withdrawing and analyzing the PhAC solution at a fixed time (3 h) and casting a fresh solution on the gel. The gel can be recycled up to seven cycles without any loss in gel consistency (Figure S4). The gel also has good removal efficiency, especially in the case of ciprofloxacin adsorption (Figure 7). This is an extremely important property, as the material can be used several times before disposal and keeps the same performance as the first cycle. It is possible to observe a gradual loss in efficiency on the fifth cycle for nalidixic acid adsorption, probably due to the saturation of adsorption sites. However, the system also works well in this case until the seventh cycle (Table S5).

This likely indicates that after the first adsorption, other adsorption sites are still available and allow HILGs to be reused without washing.

In an attempt to regenerate our sorbent systems [12,31], after the first adsorption cycle, we put the gel phases in contact with 500 μL of a sodium hydroxide solution (0.1 M) for 1.5 h. Then, we evaluated the concentration of PhAC desorbed from the gel phase, observing that 80% and 77% of the adsorbed PhAC were released from the gel to the aqueous solution for ciprofloxacin and nalidixic acid-loaded gels, respectively. However, the second adsorption cycle kept the HILG’s performance unchanged for ciprofloxacin but made the performance worse for nalidixic acid (Table S6).

Nevertheless, bearing in mind the good performance of the HILGs when no washing is performed until the seventh cycle (Figure 7), we also carried out the back washing of the resulting ciprofloxacin-laden HILG after seven adsorptions. After 6 h of contact with the aqueous NaOH solution (1 mL), we detected the desorption of 58% of the PhAC. Furthermore, in this case, the contact with a fresh sample of contaminated water catches 50% of the ciprofloxacin. This regenerability is remarkably higher than the one previously reported for some hybrid hydrogels (58% vs. 12.4%) and is achieved in shorter times (6 vs. 24 h). Indeed, in that case, full regenerability was achieved only at 95 °C [12].

The features of the PhAC-laden gels were also evaluated from a rheological point of view (Figure S5). After seven adsorption cycles, PhAC-laden gels retained their geometry and showed rheological properties even better than the fresh **graphene-G** (G′ = 1500 and 600 Pa and tan δ = 0.34 and 0.40 for **graphene-G-ciprofloxacin** and **graphene-G**, respectively).

**Figure 8:**
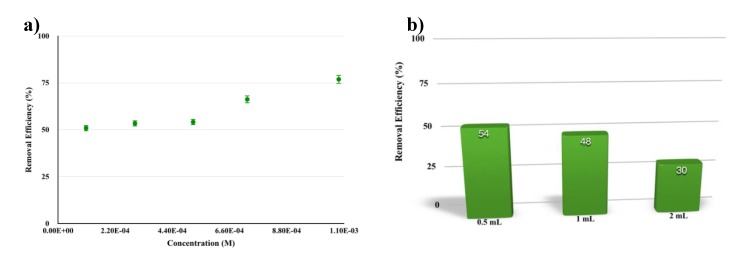
Removal efficiency of ciprofloxacin water solutions at 3 h using **graphene-G**: (**a**) as a function of ciprofloxacin concentration and (**b**) as a function of the volume of the ciprofloxacin water solution (10^−4^ M) volume on 500 mg of gel. The RE is based on triplicate runs with a reproducibility of 2%.

The adsorption process can also be influenced by the initial concentration of PhAC and the volume of the solution loaded on the gel (Figure S6). These variables were studied for ciprofloxacin adsorption after 3 h of contact with **graphene-G** (Figure 8, Tables S7 and S8).

The results obtained as a function of the PhAC concentration are in line with the literature on the adsorption of organic pollutants [18], as the removal efficiency of **graphene-G** increases when higher concentrations of ciprofloxacin are cast on the gel phase (Figure 8a). This increase is slightly higher for small variations of the concentration but passes from 50 to 75% when the concentration increases by one order of magnitude (10^−4^ compared to 10^−3^ M).

On the other hand, using the same amount of gel, the increase of the volume of the PhAC at the same concentration causes a gradual decrease in the removal efficiency of the gel (Figure 8b). Nevertheless, considering that the gel can still adsorb the PhAC, its removal efficiency in 10 mL of solution has been tested with a dialysis membrane (Figure S7). **Graphene-G** retains an RE of 27% after 24 h, indicating that the system can also work with larger volumes of wastewater. In addition, this system can maintain the total integrity of the gel inside the membrane, definitively addressing the problem of carbon nanomaterials leaching into the water after purification treatment. Similar adsorption methods have constructed the removal of paraquat and dyes in wastewater [32,33].

## 3. Materials and Methods

### 3.1. Materials

The [p-C_10_im][mal] was synthesized as previously reported in the literature [18]. The [bmim][PF_6_] was purchased from Iolitec. Before use, it was dried on a vacuum line at 60 °C for 2 h and stored in a desiccator under argon and over calcium chloride. CNTs, graphene, graphite, ciprofloxacin, nalidixic acid, and methanol were purchased from commercial suppliers and used without any further purification.

### 3.2. Gel Preparation

**Pristine-G** was prepared by weighing the right amount of gelator and IL (∼250 mg) in a screw-capped vial (diameter 1 cm). The mixture was subsequently heated and stirred at 90 °C (∼1 h). The system was, then, cooled and stored at 4 °C overnight.

To prepare the HILGs, CNTs, graphite, or graphene and the gelator were ground on an agate mortar to obtain a homogeneous powder nanocomposite. HILGs were prepared by weighing the amount of the solid mixture and IL (∼250 mg) in a screw-capped sample vial (diameter 1 cm). The mixture was first dispersed for 10 min in an ultrasonic bath (power of 200 W and frequency of 45 kHz) and subsequently heated in an oil bath at 90 °C (∼1 h) until a homogeneous dispersion of the hybrid system was obtained. In order to retain a homogeneous dispersion of CNTs in the hot solution, the system was irradiated again for 10 min before cooling the solution and storing it at 4 °C overnight. The tube inversion test method was used to examine the gel formation.

### 3.3. T*_gel_* Determination

*T*_gel_ values were determined by the falling-drop method. The vial containing the gel was immersed, turned upside down in a water and ice bath, and the bath temperature was gradually increased (2 °C min^−1^) until the first drop of the gel fell. *T*_gel_ values were reproducible within 1 °C.

Whenever possible, *T*_gel_ values were confirmed by the lead ball method. A lead ball (weighing 46.23 mg and 2 mm in diameter) was placed on top of the gel, and the vial was immersed in a water bath. The bath temperature was gradually increased (2 °C min^−1^) until the gel melted and the lead ball reached the bottom of the vial. *T*_gel_ values were reproducible within 1 °C.

### 3.4. Morphology of Gels

Pure gels at 4 wt% of the gelator and hybrid ones at 4 wt% of the gelator and 0.2 wt% of the carbon materials were cast between two glasses to record POM images. The instrument used was an Optika B-353 PL microscope equipped with crossed polarisers and an Optika camera interfaced to a computer with Optika Vision Pro Software.

For SEM analysis, the gels were washed with acetone to allow xerogels to form directly on the aluminium stubs. The dry samples, thus obtained, were shielded by gold. SEM images were recorded with an instrument with an operating voltage of 20 kV and under a low vacuum.

### 3.5. Rheological Measurements

Rheology measurements were recorded on an ARES G2 (TA Instruments) strain-controlled rheometer using a plate-plate (PP 25-2) tool. The sample was placed between the shearing plates of the rheometer. Rheological measurements, such as strain sweep and frequency sweep, were recorded three times on three different aliquots of gels. Strain sweeps were undertaken at an angular frequency of 1 rad s^−1^ and frequency sweeps at a strain of 0.025%. These values fall within the linear viscoelastic region (LVR) of gels.

For thixotropic tests, measurements were recorded at 25 °C and an angular frequency of 1 rad s^−1^, varying the strain from low (0.025%) to high percentage values (40%) for fixed time intervals of 5 min (applicable values of the yield strain were chosen based on the linear viscoelastic region and destructive strain of the gel). Rotational strain was kept at 0% for 0.05 s before changing from destructive strain to linear viscoelastic region conditions. When the moduli reached plateau values after the cessation of the disruptive strain, it was possible to calculate the initial G′ value’s percentage of recovery.

### 3.6. Thixotropic and Sonotropic Behaviors

Gels were subjected to two different external stimuli. The mechanical stimulus involved the stirring of the gel phase at 1000 rpm for 5 min using a stirring bar (length 8 mm, height 3 mm). The sonotropic behavior of the gel phases was tested via irradiation in an ultrasound water bath for 5 min with a power of 200 W and a frequency of 45 kHz. Thereafter, the materials were stored at 4 °C overnight. When the samples were stable in the tube-inversion test, the gels were defined as thixotropic or sonotropic.

### 3.7. PhAC Adsorption

The removal of PhAC was firstly tested in vials. In particular, 300 μL of a water solution with nalidixic acid or ciprofloxacin (1.8 × 10^−4^ M and 1.04 × 10^−4^ M, respectively) were cast on top of 250 mg of gels at 4% wt of the gelator and 0.2% wt of the carbon material. The RE from the aqueous solution was estimated using UV-vis spectroscopy. The final concentration was calculated according to the Beer–Lambert law (A = εbc; where A is the absorbance of the PhAC, ε is the molar extinction coefficient, where the units are mol L^−1^ cm^−1^, b is the path length of the incident light with the units cm, and c is the concentration of the PhAc in solution with the units mol L^−1^). The molar extinction coefficient of nalidixic acid (A_316 nm_) in aqueous solution was equal to 1456 mol L^−1^ cm^−1^ with b = 0.2 cm, while the one of ciprofloxacin (A_273 nm_) was equal to 7610 mol L^−1^ cm^−1^, and the final concentration of the PhAc in solution was obtained by the Beer–Lambert equation.

Recycling of the adsorbing gel was carried out by loading the suitable gel with the PhAc solution as previously described. After 3 h, the aqueous solution was removed and replaced with a fresh batch of 0.3 mL of a PhAC solution. After each cycle, the gel maintained its characteristics, as evidenced by the tube inversion test (Figure S4).

The regeneration of the gel was performed by back-washing 500 mg of gel after the adsorption of PhAC. The contact between the gel and 0.5 or 1 mL of NaOH (0.1 M) for 1.5 h or 6 h allowed the desorption of PhAC. The removal efficiency of the gels was also investigated at 24 h in a dynamic system, such as a dialysis membrane, that was filled with 1 g of gel and immersed in 10 mL of ciprofloxacin (1.04 × 10^−4^ M) water solution. Membrane dialysis was done using a cut off of 12/14,000 Dalton with a diameter of 14.3 mm.

### 3.8. Kinetics of PhAC Adsorption

The time required by gels to remove PhAC was tested with a kinetic experiment, in which 300 μL of nalidixic acid or ciprofloxacin solution (1.8 × 10^−4^ M and 1.04 × 10^−4^ M, respectively) was cast on top of 250 mg of gels at 4% wt of the gelator and 0.2% wt of the carbon material. In particular, a kinetic experiment was performed in batch using different gels for each point of the kinetic curve.

### 3.9. Determination of RE for PhAC at Different Concentrations and Volumes

Three hundred microliters of the water solution of ciprofloxacin (concentration comprised between 1 × 10^−4^ and 1 × 10^−3^ M) were cast on top of 250 mg of gels. While 0.5, 1, and 2 mL of ciprofloxacin solution (1.04 × 10^−4^ M) was cast on 500 mg of gels. Both investigations were carried out at 3 h.

## 4. Conclusions

The properties of the new HILG properties and their use as sorbents for PhAC wastewater treatment have been studied. The inclusion of carbon nanomaterials in ionic liquid gels causes an increase of 20 °C to the gel–sol transition temperature. On the other hand, the presence of CNTs reinforces the rheological properties of the gels, while graphene and graphite have a destabilizing effect on the gel’s rheological response.

Pristine and HILGs are able to adsorb PhAC from a wastewater solution without any disruption of the gel phase. This overcomes the issue of carbon nanomaterial leakage and the need to recover the material after water treatment. However, the adsorption behaviour of carbon nanomaterials trapped in a semisolid matrix is totally different from the behaviour of the free species in the solution, as soft material properties and the shape of carbon nanomaterials seem to govern the adsorption process instead of the extension of the surface area, which is the key factor for neat carbon nanomaterial adsorption processes. Indeed, **graphene-G** is the most efficient HILG and possesses the lowest stiffness and the highest self-healing ability, so it is probably more available to interact with pollutant species than **CNT-G** that features strong colloidal forces. This evidence sheds light on the presence of multiple supramolecular interactions that first occur among ionic liquid gels and carbon materials and subsequently occur among HILGs and PhAC.

**Graphene-G** is efficient (based on analyses of both PhACs), reaching 64% and 51% of removal efficiency in just 3 h. In particular, the kinetics of adsorption of ciprofloxacin is faster than that of nalidixic acid, while, at the equilibrium, the highest RE is reached for the adsorption of nalidixic acid (88%).

The adsorption system can be recycled up to seven times with a slight loss in efficiency only from the sixth to the seventh cycle. After the seventh cycle, the HILG can be washed with a small volume of sodium hydroxide solution and reused without significant loss in performance. In addition, the adsorption efficiency has also been tested in a realistic system where the HILG was embedded in a dialysis membrane. HILG are competitive with respect to single carbon nanomaterials for the adsorption of PhAC and, thanks to their recyclability, low cost, and availability, can be considered new valid alternative sorbents for this class of pollutants.

## Figures and Tables

**Table 1:** Gelation tests, critical gelation concentration (CGC), and *T*_gel_ of pristine and hybrid gels.

Entry	Gel	C (wt%)	Appearance ^b^	*T*_gel_ (°C) ^c^
1	**pristine-G [18]**	2.8 ^a^	G	SM
2		4.0	G	20
3		6.25	G	37
4	graphite-G *	3.25 ^a^	G	45
5		4.0	G	42
6		6.25	G	58
7	CNT-G *	3.25 ^a^	G	41
8		4.0	G	42
9		6.25	G	55
10	graphene-G *	3.25 ^a^	G	42
11		4.0	G	42
12		6.25	G	54

* 0.2 wt% of carbon material; ^a^ CGC; ^b^ G = gel, SM = soft material; ^c^ *T*_gel_ determined by falling drop method were reproducible within 1 °C.

**Table 2:** Elastic modulus G′, loss modulus G′′, and tan δ at angular frequency of 1 rad/s and strain of 0.025%. Error limits are based on average of three different measurements with different aliquots of gels.

Gel	G′ (Pa)	G′′ (Pa)	tan δ
**pristine-G**	3800 ± 10	1800 ± 150	0.4 ± 0.05
**CNT-G**	28,200 ± 2600	6000 ± 2000	0.23 ± 0.08
**graphite-G**	1600 ± 900	730 ± 70	0.4 ± 0.02
**graphene-G**	600 ± 100	340 ± 20	0.5 ± 0.02

**Table 3:** Removal efficiency of nanomaterial-based systems for ciprofloxacin.

Adsorbent	Time (h)	pH	RE (%)
HILG ^a^	24	7.0	58
GO [28]	24	5.0	34.5
Activated GO [29]	0.036	7.0	50
Sodium alginate/GO [30]	48	-	84.5
GO hydrogel granules [11]	120	8.0	75

^a^ This work.

## Supplementary Materials

The following are available online, Figure S1: Images of pristine and hybrid ionic liquids gels, Figure S2: POM images of hybrid gels, Figure S3: Strain and frequency sweep of HILGs, Figure S4: Images of **graphene-G** after seven cycles of PhAc adsorption, Figure S5: Strain and frequency sweep of **graphene-G** after seven cycles of PhAc adsorption, Figure S6: Images of **graphene-G** with different volumes of PhAC solution, Figure S7: Images of **graphene-G** inside a dialysis membrane in 4 mL of PhAC solution, Table S1: *T*_gel_ values at 4 wt% of gelator and variable amount of nanomaterials, Table S2: Response to external stimuli, thixotropy, and sonotropy tests, Table S3: Removal efficiency of gels after 3 h of contact with the PhAC water solution, Table S4: Kinetics of removal efficiency for both PhACs from water solutions using **graphene-G**, Tables S5 and S6: Removal efficiency of both PhACs from water solutions, at 3h, using **graphene-G** for a recycling test and after regeneration of the gel, Table S7: Removal efficiency of ciprofloxacin from water solutions, at 3h, using **graphene-G** as a function of ciprofloxacin concentration, Table S8: Removal efficiency of ciprofloxacin from water solutions, at 3 h, using **graphene-G** as function of water solution volume cast on 500 mg of gel.
