# Supplementary material for: Carbon Nanomaterial Doped Ionic Liquid Gels for the Removal of Pharmaceutically Active Compounds from Water
Source: Molecules. 2019 Jul 31;24(15):2788. doi: 10.3390/molecules24152788 (PMC6696249; doi:10.3390/molecules24152788)
Supplement: Supplementary file 1 [file molecules-24-02788-s001.pdf]

Supporting Information for

Carbon Nanomaterial Doped Ionic Liquid Gels for the Removal of Pharmaceutically Active Compounds from Water

Carla Rizzo,[1] Salvatore Marullo,[1] Nadka Tz. Dintcheva,[2] Francesca D'Anna [1,]*

[1]Università degli Studi di Palermo, Dipartimento di Scienze e Tecnologie Biologiche Chimiche e Farmaceutiche, Viale delle Scienze, Ed. 17, 90128, Palermo (Italia).

[2]Università degli Studi di Palermo, Dipartimento di Ingegneria, Viale delle Scienze Ed. 8, 90128 Palermo (Italia).

Email Corresponding Author: francesca.danna@unipa.it

Figure S1: images of pristine and hybrid ionic liquids gels. pag. S2
Figure S2: POM images of hybrid gels. pag. S2
Figure S3: strain and frequency sweep of HILGs. pag. S3
Figure S4: images of **graphene-G** after 7 cycles of PhAc adsorption. pag. S4
Figure S5: strain and frequency sweep of **graphene-G** after 7 cycles of PhAc adsorption. pag. S4
Figure S6: images of **graphene-G** with different volumes of PhAC solution. pag. S5
Figure S7: images of **graphene-G** inside a dialysis membrane in 4 mL of PhAC solution. pag. S5
Table S1: T_{gel} values at 4 wt % of gelator and variable amount of nanomaterials. pag. S6
Table S2: response to external stimuli, thixotropy and sonotropy tests. pag. S6
Table S3: removal efficiency of gels after 3 h of contact with PhAC water solution. pag. S7
Table S4: kinetic of removal efficiency of both PhACs from water solutions using **graphene-G**. pag. S7
Table S5: removal efficiency of both PhACs from water solutions, at 3h, using **graphene-G** for recycling tests. pag. S8
Table S6: removal efficiency of both PhACs from water solutions at 3h using **graphene-G** for recycling tests after regeneration of the gel. pag. S8
Table S7: removal efficiency of ciprofloxacin from water solutions, at 3h, using **graphene-G** as function of ciprofloxacin concentration. pag. S9
Table S8: removal efficiency of ciprofloxacin from water solutions, at 3h, using **graphene-G** as function of water solution volume cast on 500 mg of gel. pag. S9

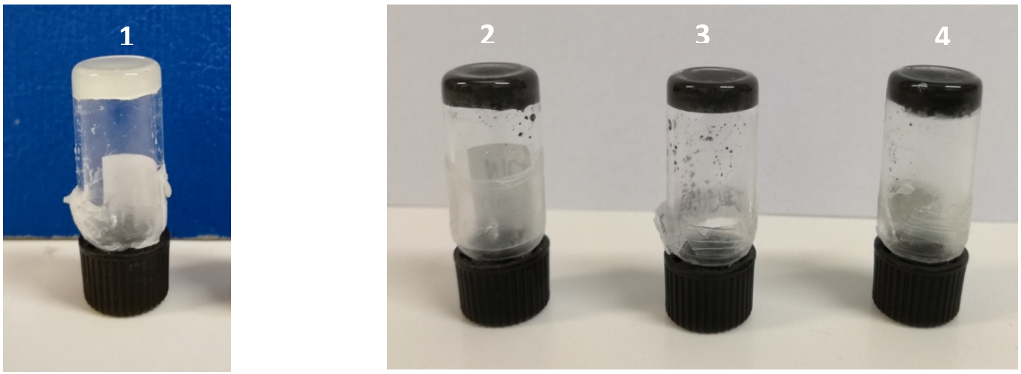

Figure S1: images of **pristine-G** (1) and hybrid ionic liquids gels: **CNT-G** (2), **graphite-G** (3), **graphene-G** (4).

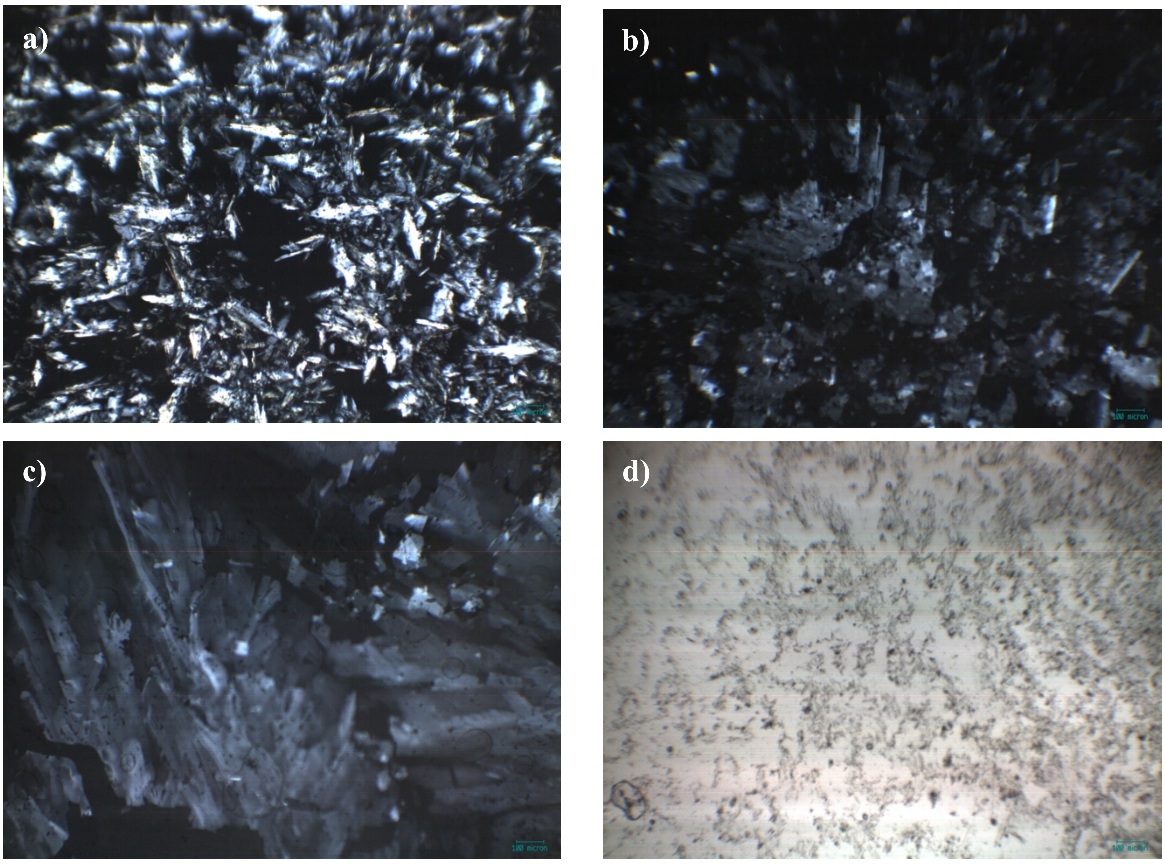

Figure S2: POM images of hybrid gels, **a) CNT-G**, **b) graphene-G**, **c-d) graphite-G**.

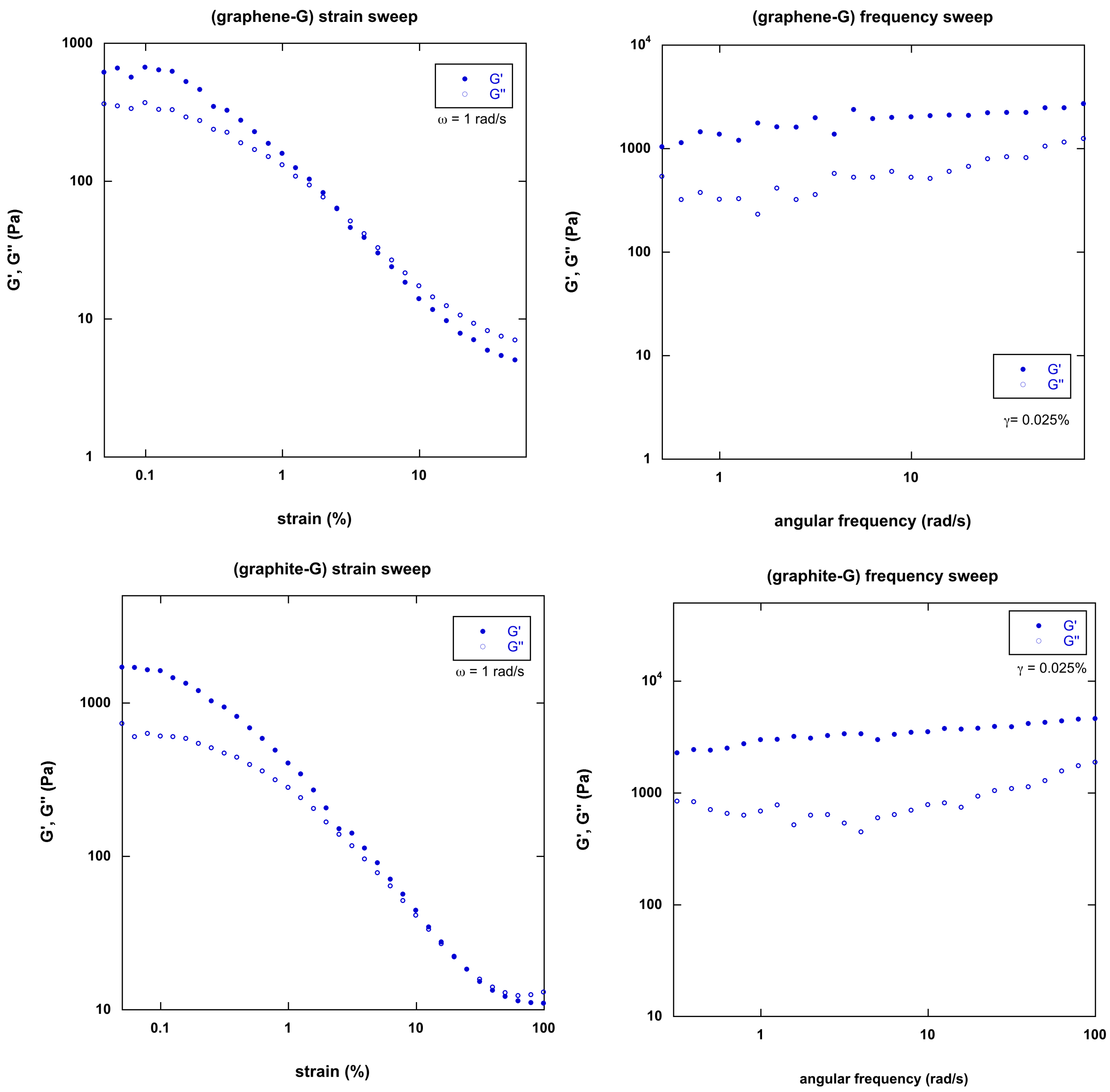

Figure S3: strain and frequency sweep of HILGs.

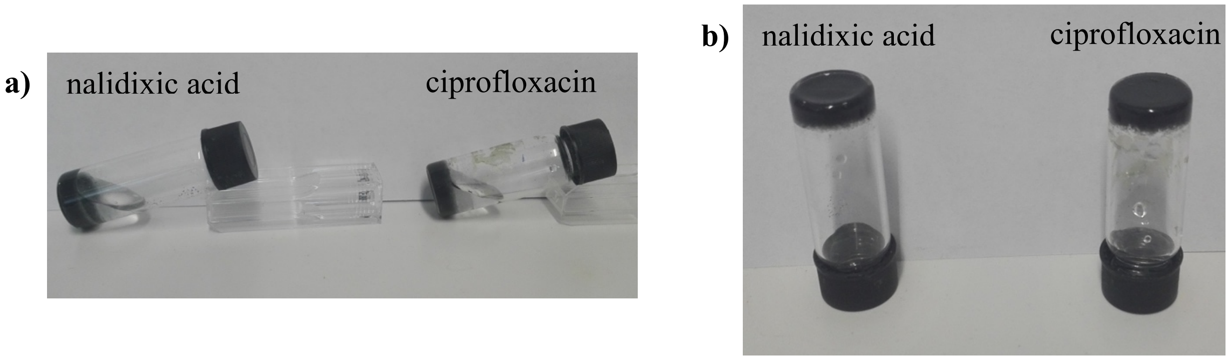

Figure S4: images of **graphene-G** after 7 cycles of PhAc adsorption.

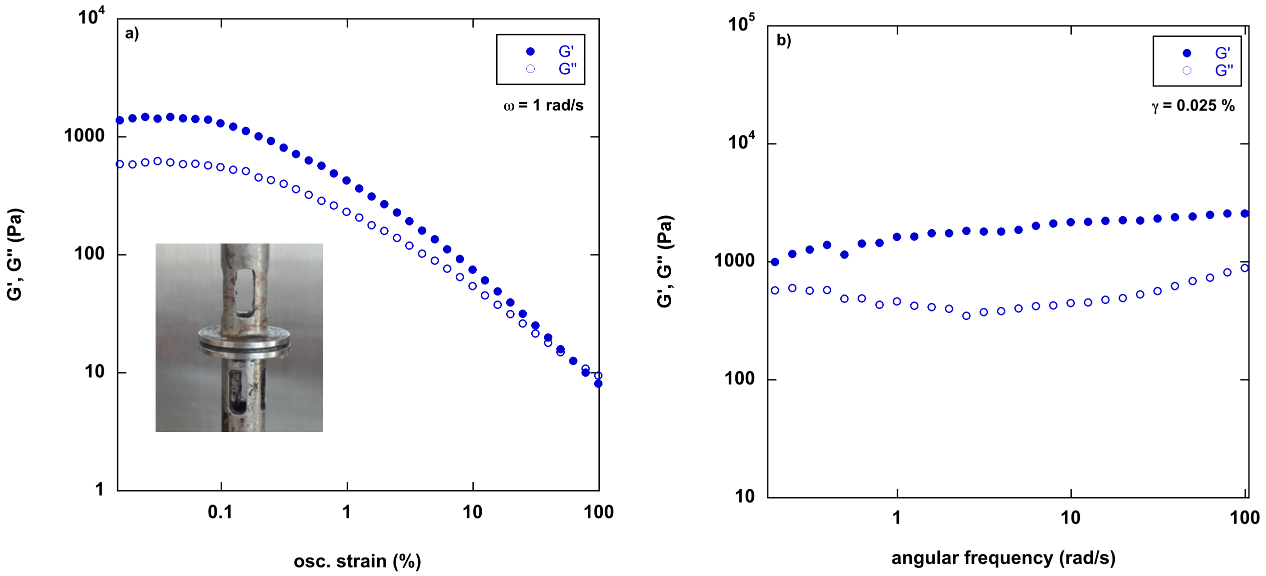

Figure S5: a) strain and **b)** frequency sweep of **graphene-G** after 7 cycles of PhAc adsorption; image of **graphene-G** keeping its geometry (inset).

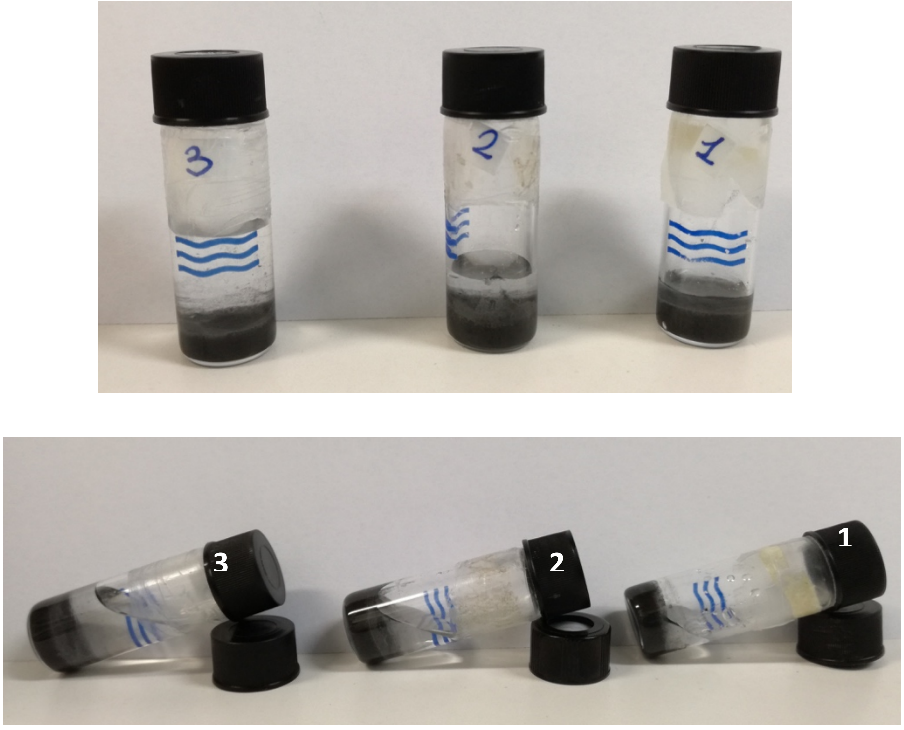

Figure S6: images of **graphene-G** (500 mg) with different volumes (vial 1, 0.5 mL; vial 2, 1 mL; vial 3, 2 mL) of ciprofloxacin water solution (10 $^{-4}$ M).

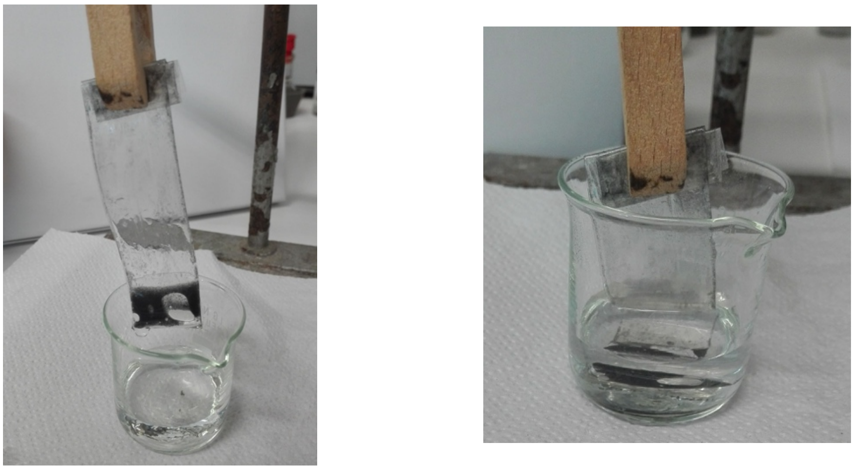

Figure S7: images of **graphene-G** (1 g) inside a dialysis membrane in 10 mL of ciprofloxacin water solution (10 $^{-4}$ M).

Table S1: T_{gel} values at 4 wt % of gelator and variable amount of nanomaterials.

Carbon Material	wt %	T_{gel} (°C)
Graphite	0.1	34
	0.2	42
	0.3	37
	0.4	35
	0.45	37
	0.6	33
Graphene	0.1	39
	0.2	42
	0.3	44
	0.4	34
	0.5	36
	0.6	34
CNT	0.1	38
	0.2	42
	0.3	39
	0.4	35
	0.5	36
	0.6	33

Table S2: response to external stimuli, sonotropy and thixotropy tests. G′ values measured during the rheological thixotropic test as function of time.

GEL	Sonotropy	Thixotropy	G′ 1° cycle	G′ 2° cycle*	G′ 3° cycle*
**pristine-G**	YES	YES	-	-	-
**graphene-G**	Stable	YES	500±100	160±60 (32%)	600±200 (100%)
**graphite-G**	Stable	NO	-	-	-
**CNT-G**	Stable	YES	26800±3400	3400±200 (13%)	2400±100 (9%)

*The percentage of strain recovery was evaluated through the comparison of G′ initial value with the one obtained in the LVR after disruption.

Table S3: removal efficiency of gels after 3 h of contact with PhAC water solution. RE is based on triplicate runs with reproducibility of 2%.

GEL	RE (%) nalidixic acid	RE (%) ciprofloxacin
**pristine-G**	61	49
**CNT-G**	49	50
**graphene-G**	64	50
**graphite-G**	59	50

Table S4: kinetic of removal efficiency of both PhACs from water solutions using **graphene-G**. RE is based on triplicate runs with reproducibility of 2%.

Time (h)	RE (%) nalidixic acid	Time (h)	RE (%) ciprofloxacin
0.12	14	0.12	30
0.25	27	0.25	34
0.5	36	0.5	44
1	41	1	42
2	51	2	45
3	61	3	51
4	61	4	57
5	70	5	52
6	69	6	57
15	81	15	57
24	88	24	58

Table S5: removal efficiency of both PhACs from water solutions at 3h using **graphene-G** for recycling tests. RE is based on triplicate runs with reproducibility of 2%.

	**RE (%) nalidixic acid**	**RE (%) ciprofloxacin**
**I cycle**	64	51
**II cycle**	65	47
**III cycle**	70	45
**IV cycle**	67	50
**V cycle**	53	50
**VI cycle**	57	53
**VII cycle**	55	54
**VIII cycle**	31	29

Table S6: removal efficiency of both PhACs from water solutions at 3h using **graphene-G** for recycling tests after regeneration of the gel. RE is based on triplicate runs with reproducibility of 2%.

	**RE (%) nalidixic acid**	**RE (%) ciprofloxacin**
**1° adsorption**	64	51
**1° desorption**	77	80
**2° adsorption**	40	49

Table S7: removal efficiency of ciprofloxacin from water solutions at 3h using **graphene-G** as function of ciprofloxacin concentration. RE is based on triplicate runs with reproducibility of 2%.

Concentration (M)	RE (%) ciprofloxacin
$1.08 \cdot 10^{-4}$	51
$2.96 \cdot 10^{-4}$	53
$5.20 \cdot 10^{-4}$	54
$7.28 \cdot 10^{-4}$	66
$1.08 \cdot 10^{-3}$	77

Table S8: removal efficiency of ciprofloxacin (10^{-4} M) from water solutions, at 3h, using **graphene-G** as function of water solution volume cast on 500 mg of gel. RE is based on triplicate runs with reproducibility of 2%.

Volume	RE (%) ciprofloxacin
0.5 mL	54
1 mL	48
2 mL	30
